# Measurement and prediction on thermal conductivity of fused quartz

**DOI:** 10.1038/s41598-020-62299-y

**Published:** 2020-04-16

**Authors:** Xin Rui Zhang, Gang Qiang Kong, Le Hua Wang, Xiao Liang Xu

**Affiliations:** 1 Key Laboratory of Ministry of Education for Geomechanics and Embankments, Hohai University, Nanjing, P.R. China; 2 Key Laboratory of Geological Hazards on Three Gorges Reservoir Area of Ministry of Education, China Three Gorges University, Yichang, P.R. China

**Keywords:** Materials science, Theory and computation, Computational methods

## Abstract

Thermal conductivity of soil is a basic physical property related to heat conduction, and also is one of parameters widely applied in geotechnical engineering. The effect of gradation on the thermal conductivity of fused quartz was analyzed by thermal needle tests. The different particle size with the same uniformity coefficient (*C*_u_ = 3.2) and different uniformity coefficient for the same particle size (0.10~1.00 mm) were considered in this study. It shows that the thermal conductivity of fused quartz decreases with the decreasing of the mean particle size and with the increasing of the porosity. Simple modified methods to estimate the value of thermal conductivity are proposed, and had been demonstrated successfully by conducting fused quartz, carbonate sand and Ottawa sand.

## Introduction

Management of natural resources is becoming one of the crucial issues of the 21st Century. The global demand for clean and renewable energy sources is growing because they represent one of the most effective tools against climate change. As a kind of renewable energy, shallow geothermal energy not only has huge reserves, no pollution and no carbon emissions, but also is easy to develop^1–3^. In order to save underground space, geotechnical engineers combine ground source heat pump technology with underground structures (such as pile, tunnel, etc.) as energy geological structures. It is believed that the energy geological structure has made a positive contribution to regulating the environmental conditions of the building. The working process of energy geostructures is a coupling problem of stress field, displacement field, temperature field, and seepage field. The efficiency of heat transfer affects the efficiency of energy geostructures. Thermal conductivity of soil is a basic physical property related to heat conduction.

Transparent soil consists of an aggregate and a refractive index matched fluid^4^. When fully saturated, the particles appear invisible and allow light to pass, enabling visualization through the “soil”^4^. Both fine (clay) and coarse-grained (sands/gravels) materials were developed, and their mechanical properties were summarized by Iskander *et al*.^5^. Combined with laser and particle image velocimetry, non-invasive measurements can be made to investigate internal flow, deformation of transparent soil around the structure, grouting, and thermal reponse of sands in geotechnical engineering^6^. The heat transfer processes of energy pile surrounding soil were measured through transparent soil^7,8^. However, previous literature has not studied the thermal properties of fused quartz and its difference in thermal behavior from that of natural soils. Measured thermal conductivity of fused quartz will also be beneficial to future studies on thermal reponses of granular materials using transparent sand.

Thermal conductivity refers to the heat flow per unit area per unit time in a unit temperature gradient (with unit: W/(m·K)). It depends on the mineral composition, water content, dry density, particle composition and morphology, temperature, etc.^9,10^. De Vries^11^ introduced soil particle shape coefficient *g*_s_ in his model of heat transfer. Tarnawski *et al*.^12^ provided that particle shape values (*g*_s_) for C-109 is 0.12, and for C-190 is 0.125. Rzhevsky *et al*.^13^ found that the thermal conductivity decreased as the particle size of the material decreases. Midttomme *et al*.^14^ found that the particle content > 63 μm had the greatest effect on the measured thermal conductivity. Xiao *et al*.^15^ found that the gradation effect on the thermal conductivity is attributed to the same intrinsic mechanism, which is independent of the porosity. An important internal factor that has not been fully investigated is the effect of gradation, which is a relevant topic in the study of application of transparent soils. Hence, a series of laboratory tests were performed to investigate the influence of the gradation and porosity on the thermal conductivity of the fused quartz. This study reviews literature models for the thermal conductivity of granular materials, experimentally determines the thermal conductivity of fused quartz using the thermal needle probe method, and assesses the accuracy of predicting the measured data using the existed models. The results hightlight the inadequacy of these models in characterizing the measured thermal conductivity of fused quartz and thus a new fitting formula based on literature models is suggested.

## Materials and methods

### Fused quartz

Transparent soils are produced by mixing the fused quartz and a refractive index matched pore fluid^4^. The fused quartz has similar geotechnical properties to the nature sand and the fused quartz is highly angular and possesses chemical composition similar to that of natural silicate sand^5^. The main advantages of transparent soil over previous recipes include lower pore fluid viscosity, lower fluid sensitivity to temperature variations, and the ability to recycle the materials for use in multiple tests^5^. More details on the physical and mechanical properties of the fused quartz can be found in the literatures^5,6,16–18^.

**Figure 1:**
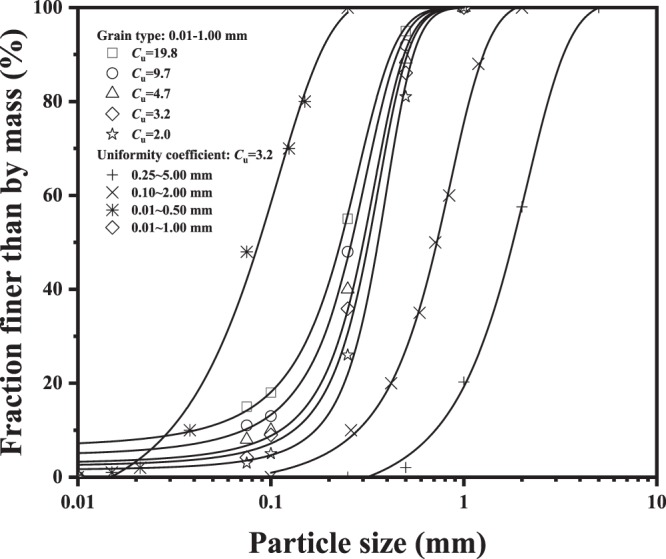
Gradations of the fused quartz used in this study.

In this paper, the fused quartz with a purity of 99.9%, manufactured by Xuzhou Xinyi Wanhe Minerals Co., Ltd., China, was chosen as transparent granular particles to mimic natural sand particles^6,19^. This fused quartz is a kind of angular uranium glass particles, owing to crushing of the production process. According previous application study of transparent soil^7,8^, four difference particle sizes with 0.50~1.00 mm, 0.01~1.00 mm, 0.01~2.00 mm, and 0.25~5.00 mm were used in this experiment, which can obtain by sieving and mixing, as shown in Figure 1. The specific gravity of the fused quartz is 2.14. Previous literature shows that the relationships between thermal conductivity (*λ*) and uniformity coefficients (*C*_u_ = *d*_60_/*d*_10_) for different porosity are parallel, indicating that the gradation effect on the thermal conductivity is attributed to the same intrinsic mechanism, which is independent of the porosity^15^. Hence, uniformity coefficient (*C*_u_ = 3.2) for different particle size was also considered for the fused quartz in this study. Basic properties of these samples are shown in Table 1.

**Table 1:** Basic properties of fused quartz samples with different situation.

Grain range (mm)	*C*_u_	*d*_*50*_(mm)	*ρ*_dmax_ (g/cm^3^)	*ρ*_dmin_ (g/cm^3^)	Porosity	*S*_r_
0.01~1.00	19.8	0.36	1.383	1.148	0.40, 0.42, 0.44, 0.46	0
0.01~1.00	9.7	0.32	1.371	1.123	0.40, 0.42, 0.44, 0.46	0
0.01~1.00	4.7	0.30	1.365	1.106	0.40, 0.42, 0.44, 0.46, 0.48	0
0.01~1.00	3.2	0.26	1.343	1.093	0.40, 0.42, 0.44, 0.46, 0.48	0
0.01~1.00	2.0	0.23	1.327	1.08	0.40, 0.42, 0.44, 0.46, 0.48	0
0.25~5.00	3.2	1.74	1.463	1.162	0.36, 0.38, 0.40, 0.42, 0.44	0
0.10~2.00	3.2	0.72	1.409	1.098	0.42, 0.44, 0.46, 0.48	0
0.01~0.50	3.2	0.09	1.27	1.054	0.42, 0.44, 0.46, 0.48	0
0.01~1.00	3.2	0.26	1.343	1.093	0.40	0.3, 0.5, 0.8, 1.0
0.01~1.00	3.2	0.26	1.343	1.093	0.42	0.3, 0.5, 0.8, 1.0
0.01~1.00	3.2	0.26	1.343	1.093	0.44	0.3, 0.5, 0.8, 1.0

*Notes*: *C*_u_ is the uniformity coefficient, *d*_50_ is mean particle size of the fused quartz, *ρ*_dmax_ is the maximum dry density, *ρ*_dmin_ is the minimum dry density and *S*_r_ is the saturation.

### Testing methods

Steady state method and transient method are the two main test principles of thermal properties measurement. In this study, the KD2 Pro thermal properties analyzer developed by METER Group, Inc. USA, was used as the test instrument, which belongs to transient method^15^. The KD2 Pro thermal properties analyzer is widely utilized as a tool to research thermal conductivity of soil^15,20,21^. The test probe is selected as the SH-1 probe with an accuracy of 5%, and 30 mm length. The measurement medium with a thickness of 1.5 cm or more in any direction around the sensor is guaranteed. More information about the instrument may be seen in the KD2 Pro Manual. The soil sample was tested with a diameter of 50 mm and a height of 70 mm. The specimen preparation and experimental procedures are as follows:

1. The fused quartz was placed in an oven setting temperature of 105 °C for 24 h, then sands were permitted to cool before sieving to different gradations. Maximum and minimum dry density of the fused quartz with the different particle size are shown in Table 1, measuring based on ASTM D4253 (ASTM 2016*a*) and ASTM D4254 (ASTM 2016*b*).
2. Specimens with different porosity and the uniformity coefficients are shown in Table 1 and were prepared according to the four particle sizes in Figure 1. The under compaction method proposed by Ladd^22^ was used to obtain uniform specimens. Five layers were controlled, and a layer forms with its compacted dry density slightly greater (approximately 1%) than that of the substratum layer^15^. Compaction was not observed to further alter the gradations.
3. The SH-1 probe was inserted into the center position of the specimen using a guide to ensure verticality. The thermal conductivity of the fused quartz species was measured by the KD2 Pro thermal properties analyzer. Although a slight densification of the fused quartz is possible during needle insertion, measurements of the three specimens of 0.01~1.00 mm (*C*_u_ = 3.2) for n = 0.40 were within a ±2% range of the average value of measured thermal conductivities reported in Table 2, which indicates that this approach is repeatable. The thermal conductivity of the fused quartz was measured under controlled room-temperature conditions (25 °C ± 1 °C) to minimize the influence of the ambient temperature.

**Table 2:** Test results on thermal conductivity of fused quartz.

Grain type (mm)	*C*_u_	Porosity	*λ* (W/m^−1^K^−1^)	Grain type (mm)	*C*_u_	Porosity	*λ* (W/m^−1^K^−1^)
0.01~1.00	2.0	0.40	0.183	0.01~1.00	19.8	0.40	0.212
		0.42	0.176			0.42	0.205
		0.44	0.173			0.44	0.201
		0.46	0.166			0.46	0.197
		0.48	0.160			—	—
0.01~1.00	3.2	0.40	0.186/0.189/0.191	0.25~5.00	3.2	0.36	0.212
		0.42	0.183			0.38	0.204
		0.44	0.177			0.40	0.197
		0.46	0.172			0.42	0.189
		0.48	0.167			0.44	0.185
0.01~1.00	4.7	0.40	0.198	0.10~2.00	3.2	0.42	0.186
		0.42	0.193			0.44	0.181
		0.44	0.185			0.46	0.174
		0.46	0.183			0.48	0.170
		0.48	0.177			—	—
0.01~1.00	9.7	0.40	0.208	0.01~0.50	3.2	0.42	0.178
		0.42	0.203			0.44	0.173
		0.44	0.196			0.46	0.166
		0.46	0.191			0.48	0.162
		—	—			—	—

*Notes*: λ is the thermal conductivity.

## Results and analysis

### Experimental results of thermal conductivity of the fused quartz

**Figure 2:**
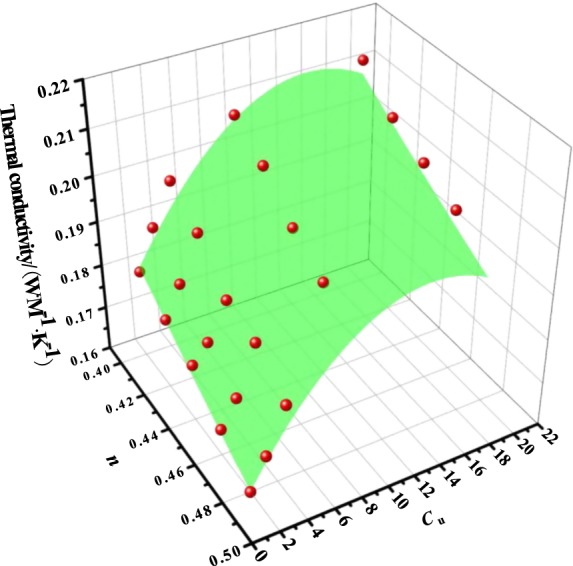
Relationships among the porosity and the thermal conductivity of fused quartz in different uniformity coefficient.

The relationships between thermal conductivity and porosity for the different *C*_u_ are parallel in Figure 2. It indicates that the gradation effect on the thermal conductivity is attributed to the same intrinsic mechanism, which is independent of the void ratio. Figure 2 shows that the thermal conductivity at a given porosity increased with the increasing of uniformity coefficient (*C*_u_) when 2.0 < *C*_u_ ≤ 4.7, and increased gradually when *C*_u_ > 4.7. Similar results are also obtained for Carbonate sand^15^.

**Figure 3:**
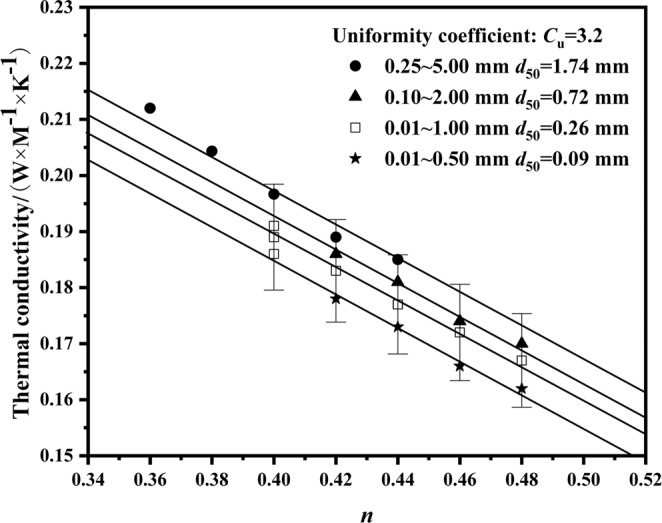
Relationships between thermal conductivity of fused quartz and porosity in different grain type.

**Figure 4:**
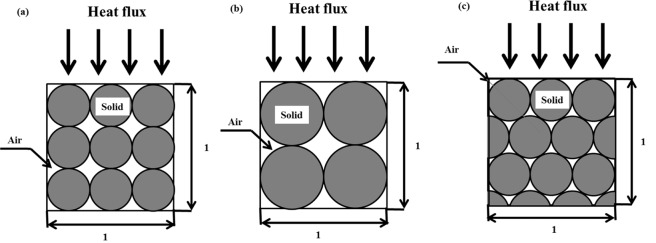
Soil particles per unit volume in ideal arrangement.

Relationships between thermal conductivity of the fused quartz and porosity in different grain type are shown in Figure 3. In order to preserve clarity and avoid overlapping, the error bars (within the precision range of ±5% of the KD2 Pro thermal properties analyzer) were applied to one set of data. It shows that the thermal conductivity decreases with the increase of porosity, which is in accordance with the results reported by Luikov^23^, Baldi *et al*.^24^, Côté & Konrad^25^, and Barry-Macaulay *et al*.^26^. Figure 3 shows that the thermal conductivity increased with the increasing of the mean particle size with the same uniformity coefficient which is same with the founding of Rzhevsky *et al*.^13^ For explanation of trend change in Figure 3, soil elements were simplified as follow Figure 4. When heat flows in Figure 4(a), the number of heat transfer paths from solid to air is more than in Figure 4(b). Heat transfer paths of Figure 4(b) in continuous solid are more than Figure 4(a). So the thermal conductivity increased with the increasing of the mean particle size, as shown in Figure 3. For the decrease of porosity, it is like Figure 4(a) to (c). There are more solid particles in the same volume. Thermal conductivity of fused quartz is greater than that of air. With the increasing of porosity, air content of soils increased. Then the thermal conductivity of fused quartz decreased^27^.

The fitting lines show the relationship between thermal conductivity with porosity for different grain type in Figure 3. This simple linear function^28^ describes the relationship between *λ*_dry_ and *n* for mineral soils:

1 $$\lambda =c+dn$$

where *c* and *d* are the empirical parameters, depending on the textures, quartz contents and mineral composition, and the value range of *n* depends on the maximum and minimum voids that fused quartz can reach to. Eq. (1) was provided by Lu *et al*.^28^ based on Johansen^29^ data and heat pulse data. In this literature, *c* = 0.51 and *d* = -0.56 for 0.2 < *n* < 0.6. After that He *et al*.^30^ also adopted this linear model and proposed different parameters: *c* = 0.50 and *d * = -0.58 for a wide range of textures and quartz contents, which are similar with Lu *et al*.^28^, while *c* = 1.18 and *d* = -1.92 for calculating the thermal conductivity of sands with >99% quartz contents. Based on these two literatures, the values of *c* and *d* depend on the textures and quartz contents. Since fused quartz is different from traditional soils, the values of *c* and *d* are different from literatures. For fused quartz, *d* = -0.3, shown in Figure 3.

### Prediction of the thermal conductivity of fused quartz

The root relative mean square error (*RRMSE*) as show as Eq. (2) is used to evaluate the results of calculation using literature models in Table 3.

2 $$RRMSE=\sqrt{\frac{1}{N}{\sum }_{1}^{N}{\left(\frac{{\lambda }_{\exp }-{\lambda }_{cal}}{{\lambda }_{\exp }}\right)}^{2}}\times 100 \% $$

where λ_exp_ is the experimental value, and λ_cal_ is the calculative value.

**Table 3:** *RRMSE* (%) of predicted thermal conductivity of fused quartz using modified models.

Grain type (mm)	0.01~1.00					0.25~5.00	0.10~2.00	0.01~0.50
*C*_u_	2.0	3.2	4.7	9.7	19.8	3.2		
Modified Vincent model (Vincent *et al*.^26^)	4.26	5.01	4.94	3.77	4.32	4.12	3.51	3.34
Modified Tong model (Tong *et al*.^13^)	8.08	8.00	6.91	6.04	5.88	4.10	5.50	8.25
Modified Johansen model (Johansen^24^)	3.24	3.86	3.81	2.79	3.32	2.87	4.13	2.91
Vincent model (Vincent *et al*.^26^)	35.23	36.35	40.64	42.63	43.83	36.36	39.34	36.48
Tong model (Tong *et al*.^18^)	12.31	13.82	19.51	22.05	23.71	13.67	17.67	13.84
Johansen model (Johansen^24^)	32.34	30.74	21.77	17.68	15.53	31.59	23.40	29.10

#### Prediction of the thermal conductivity of fused quartz using literature model

Johansen^29^ improved De Vries^11^ model, and then proposed a semi-empirical relationship to predict the thermal conductivity of dry soils,

3 $${\lambda }_{dry}=\frac{0.135{\rho }_{d}+64.7}{{\rho }_{s}-0.947{\rho }_{d}}$$

4 $$n=1-\frac{{\rho }_{d}}{{\rho }_{s}}$$

where *ρ*_d_ is the dry density of soils (kg/m^3^), and *ρ*_s_ is the soil particle density (kg/m^3^), *A* = 0.135, *B* = 64.8 and *C* = 0.947. For the fused quartz, *ρ*_s_ = 2140 kg/m^3^. Eq. (4) can be applies for dry, unsaturated and saturated soil. The values of RRMSE almost are more than 20%. Based on De Vries^11^ model, coefficients in Eq. (3) are shown as follow,

5 $$A={\lambda }_{a}\cdot ({k}_{s}\cdot {\lambda }_{s}/{\lambda }_{a}-1)$$

6 $$B={\lambda }_{a}\cdot {\rho }_{s}$$

7 $$C=1+{k}_{s}$$

8 $${k}_{s}=1/3[2/(1+({\lambda }_{s}/{\lambda }_{a}-1){g}_{s})+1/(1+({\lambda }_{s}/{\lambda }_{a}-1)(1-2{g}_{s}))]$$

where *λ*_s_ is the thermal conductivity of solid, *λ*_a_ is the thermal conductivity of air, *k*_s_ is the weighting shape factor, and it is usually treated as a fitting parameter^12^, and *g*_s_ is shape value of soil particles. The reason for poor fitting effect using Eq. (3) is improper coefficients values.  

Vincent^31^ proposed the equation to predict the thermal conductivity of dry soils,

9 $${\lambda }_{dry}=\frac{(p{\lambda }_{s}-{\lambda }_{a})\times {\rho }_{d}+{\lambda }_{a}{G}_{s}}{{G}_{s}-(1-a)\times {\rho }_{d}}$$

where *G*_s_ is the specific gravity, and *p* is the empirical parameter, *p* = 0.053. Note that this equation is the same as Eq. (3) when *λ*_s_ = 3 W•m^−1^•K^−1^, *λ*_a_ = 0.024 W•m^−1^•K^−1^, and *ρ*_d_ = 2.7 g•cm^−3^. For the fused quartz, substitute Eq. (4) into Eq. (9), and *λ*_s_ = 1.4 W•m^−1^•K^−1^ ^32^. The values of *RRMSE* are more than 30% that is not enough for the accuracy of predicting the thermal conductivity of fused quartz. The reason for poor fitting effect using Eq. (9) is improper coefficients value.

Substitute Eq. (4), (5), (6) and (7) into Eq. (3), we can get,

10 $${\lambda }_{dry}=\frac{({k}_{s}{\lambda }_{s}-{\lambda }_{a})(1-n)+{\lambda }_{a}}{1-(1-{k}_{s})(1-n)}$$

Substitute Eq. (4) into Eq. (9), we can get,

11 $${\lambda }_{dry}=\frac{(p{\lambda }_{s}-{\lambda }_{a})(1-n)+{\lambda }_{a}}{1-(1-p)(1-n)}$$

where *p*, just like *k*_s_, is the weighting shape factor, and it is usually treated as a fitting parameter at Eq. (9).  

Tong *et al*.^33^ improved Wiener model, and then proposed a semi-empirical semi-theoretical model to predict the thermal conductivity of dry soils.

12 $${\lambda }_{dry}={\eta }_{1}(1-n){\lambda }_{s}+{\eta }_{1}n{\lambda }_{a}+(1-n)\frac{{\lambda }_{a}{\lambda }_{s}}{n{\lambda }_{s}+(1-n){\lambda }_{a}}$$

where parameter *η*_1_ usually can be obtained by a series of experiments that require the samples of different porosities but with zero saturation (dry samples), and Tong provide an empirical equation about *η*_1_ considering Johansen model^29^. In a sense, parameter *η*_1_ describes the way of contact between soil particles.

13 $$p=0.0692{n}^{-0.7831}$$

For fused quartz, *λ*_s_ = 1.4 W•m^−1^•K^−1^. The values of *RRMSE* are more than 10% which is not enough for the accuracy of predicting the thermal conductivity of fused quartz. Obviously Eq. (13) was obtained by Eq. (3), it is not surprising that the fitting effect of Eq. (13) is poor for fused quartz.

#### Modified literature models

**Figure 5:**
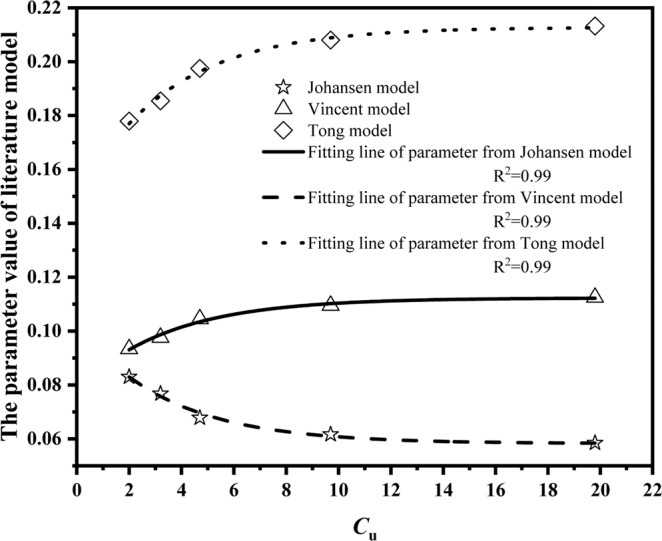
The parameter value of literature model versus *C*_u_ for fused quartz.

For Johansen model, Vincent model and Tong model, parameter can be modified as follow:

14 $${\eta }_{1}={k}_{s}=p=a-b\times {0.75}^{{C}_{u}}$$

where for Johansen model *a* = 0.06 and *b* = –0.04, for Vincent model, *a* = 0.11 and *b* = 0.03, and for Tong model, *a* = 0.21 and *b* = 0.06, shown in Figure 5. Eq. (14) provides a relationship for the effect of particle composition on thermal conductivity. In this study, *a* and *b* are empirical parameters. *a* is the maximum value of the coefficient, while *b* control the rate of change of the coefficient.

**Figure 6:**
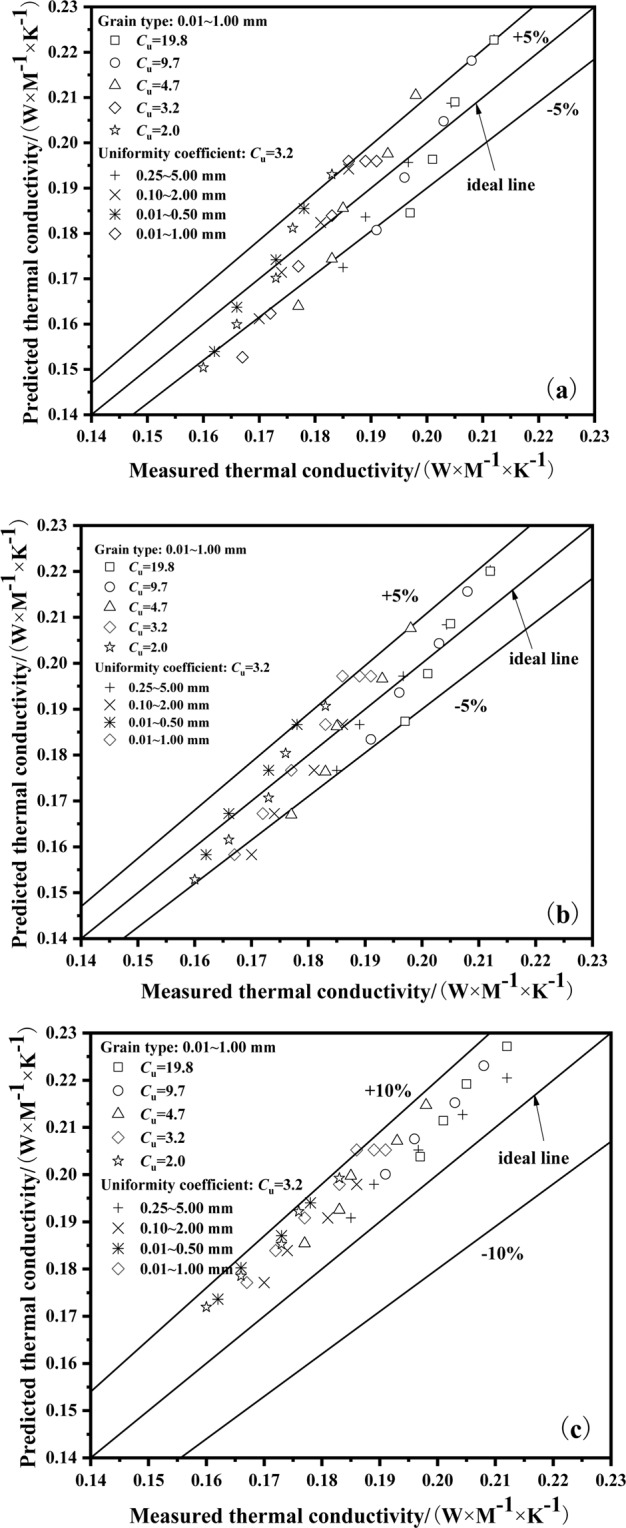
Predicted thermal conductivity values versus measured values using (**a**) modified Vincent model ; (**b**) modified Johansen model; and (**c**) the modified Tong model.

Figure 6 presents the comparison between predicted thermal conductivity using (a) modified Vincent model; (b) modified Johansen model; and (c) the modified Tong model and measured thermal conductivity of the fused quartz. It is evident that the predicted values compare well with the measured values for the modified Vincent model and modified Johansen model, almost in the ±5% range, and the modified Tong model is higher than measured thermal conductivity in the ±10% range.

The values of root relative mean square error using modified Johansen model, modified Vincent model and modified Tong model are shown in Table 3. It shows that for modified Johansen model the values of *RRESM* are between 2% and 5%, for modified Vincent model the values of *RRESM* are between 3% and 5%, and for modified Tong model the values of *RRESM* are almost between 4% and 9%.

#### Modified literature models for literature data

**Figure 7:**
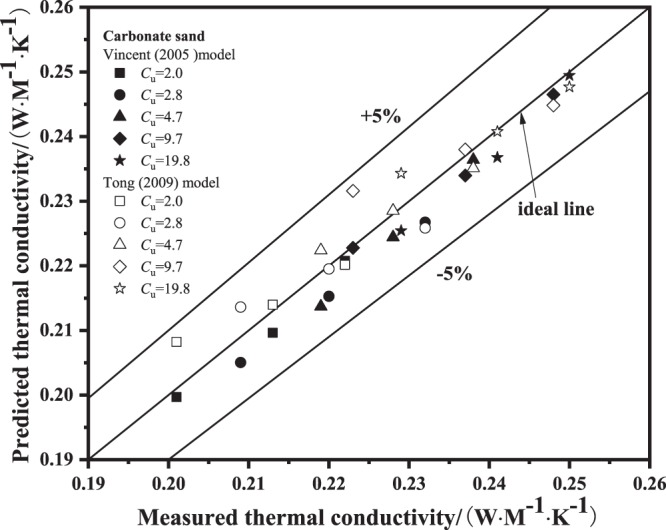
Predicted thermal conductivity values using modified models versus Carbonate sand^15^.

The thermal conductivity of carbonate sand^15^ was predicted by using the modified Vincent model and modified Tong model: for Vincent model, *a* = 0.038 and *b* = 0.09, while for Tong model, *a* = 0.071 and *b* = 0.018. Figure 7 presents the comparison between predicted thermal conductivity using the modified Vincent model and modified Tong model and measured thermal conductivity of carbonate sand. It is evident that the predicted values compare well with the measured values, in the ±5% range. The values of root relative mean square error using modified Vincent model and modified Tong model are shown in Table 4. The values of *RRESM* of predicted thermal conductivity of Carbonate sand^15^ using modified models are between 0% and 3%.

**Table 4:** *RRMSE* (%) of predicted thermal conductivity of Carbonate sand^15^ using modified models.

*C*_u_	2.0	2.8	4.7	9.7	19.8
Modified Vincent model (Vincent *et al*.^26^)	1.04	2.11	1.71	0.81	1.36
Modified Tong model (Tong *et al*.^13^)	2.14	2.00	1.14	2.36	1.44

**Figure 8:**
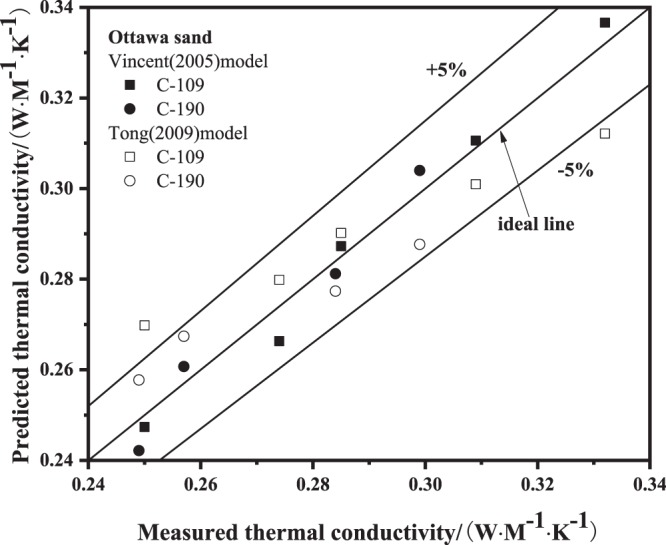
Predicted thermal conductivity values using modified models versus Ottawa sand^12^.

The thermal conductivity of Ottawa sand^12^ was also predicted by using the modified Vincent model and modified Tong model: for Vincent model, *a* = 0.029 and *b* = 0.01, while for Tong model, *a* = 0.099 and *b* = 0.077. Figure 8 presents the comparison between predicted thermal conductivity using the modified Vincent model and modified Tong model and measured thermal conductivity of Ottawa sand. It is evident that the predicted values compare well with the measured values, in the ±5% range. The values of root relative mean square error of Ottawa sand C-109 using modified Vincent model and modified Tong model are 1.54% and 4.76%, respectively. The values of root relative mean square errors of Ottawa sand C-190 using modified Vincent model and modified Tong model are 1.83% and 3.48%, respectively. The values of *RRESM* of predicted thermal conductivity of Ottawa sand using modified models are between 0% and 5%.

#### Modified Tong model for the three-phase mixture

**Figure 9:**
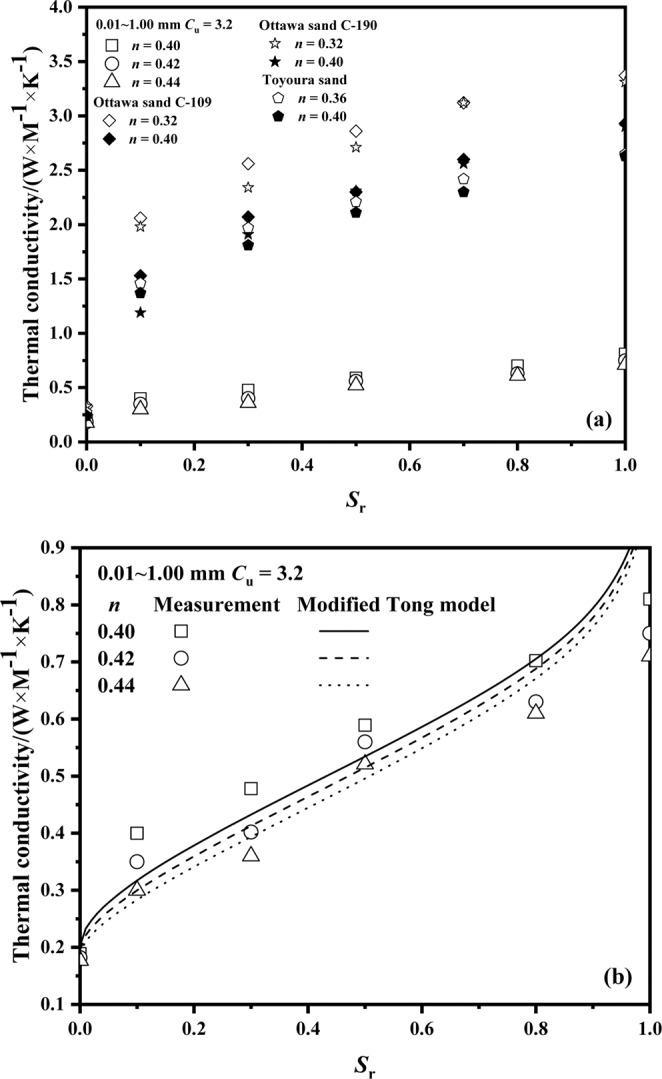
Thermal conductivity versus saturation of (**a**) comparison between fused quartz and natural sands^12^; and (**b**) fused quartz.

In order to verify the new modified Tong model, a set of samples were tested at different degrees of saturation from dryness to full saturation. The measurement results are shown in Figure 9(a). The values of thermal conductivity for natural sands are higher than that of fused quartz due to the difference in minerals. At a low *S*_r_ range, 0 < *S*_r_ < 0.1, a sharp increase by about 100% with *S*_r_ was observed, while for 0.1 < *S*_r_ < 1, the rate of linear increase was about 100%.

Tong *et al*.^33^ also proposed a semi-empirical semi-theoretical model to predict the thermal conductivity of the three-phase.

15 $$\begin{array}{c}\lambda ={\eta }_{1}(1-n){\lambda }_{s}+(1-{\eta }_{2}){[1-{\eta }_{1}(1-n)]}^{2}\times \\ {\left[\frac{(1-n)(1-{\eta }_{1})}{{\lambda }_{s}}+\frac{n{S}_{r}}{{\lambda }_{w}}+\frac{n(1-{S}_{r})}{{\lambda }_{g}}\right]}^{-1}\\ +{\eta }_{2}[(1-n)(1-{\eta }_{1}){\lambda }_{s}+n{S}_{r}{\lambda }_{w}+n(1-{S}_{r}){\lambda }_{g}]\end{array}$$

where parameter *η*_2_ should be a function of porosity and saturation, *η*_2_ =${0.59S}_{r}^{1.487n-0.0404}$.

The calculation results using modified Tong model are shown in the Figure 9(b). *RRMSE* (%) of predicted thermal conductivity of fused quartz from dryness to full saturation using modified the Tong model modified and Tong model is shown in Table 5. For each porosity, the values of RRMSE reduced by 4%.

**Table 5:** *RRMSE* (%) of predicted thermal conductivity of fused quartz from dryness to full saturation using the Tong model modified and Tong model.

*n*	0.40	0.42	0.44
Tong model	20.09	19.38	19.25
Modified Tong model	16.03	15.30	15.33

## Conclusions

In this study, the thermal conductivity of fused quartz is first measured, and three empirical predicted models are modified for calculating the thermal conductivity of fused quartz. Nearly 100 test results from previous literature are used to verification the modified empirical predicted models. Following conclusions can be drawn:

1. The thermal conductivity at a given porosity increases with the increasing of the uniformity coefficient when 2.0 < *C*_u_ ≤ 4.7, and it increases gradually when *C*_u_ > 4.7. The thermal conductivity decreases with the increasing of the porosity, and increased with the increasing of the mean particle size with the same uniformity coefficient.
2. Three modified models (Vincent model, Tong model, and Johansen model) can be used for predicting the thermal conductivity of fused quartz with acceptable precision. A simple modified method to estimate the value of thermal conductivity has been proposed, and its utility and efficiency have been demonstrated successfully by conducting the fused quartz, carbonate sand and Ottawa sand.
